# Stanford Type B aortic dissection following transradial coronary angiography in a patient with an aberrant right subclavian artery: a case report

**DOI:** 10.3389/fcvm.2026.1910957

**Published:** 2026-08-24

**Authors:** Jun Ma, Wei Wang, Hua Yan

**Affiliations:** Department of Cardiology, Wuhan Asia Heart Hospital, Wuhan, China

**Keywords:** aberrant right subclavian artery, transradial coronary angiography, iatrogenic Stanford Type B aortic dissection, vascular complication, conservative treatment

## Abstract

Aberrant right subclavian artery (ARSA) represents the most prevalent congenital anatomical malformation of the aortic arch. Most affected patients remain asymptomatic, making ARSA prone to misdiagnosis before procedures, which markedly increases the risk of vascular injury during interventional procedures through the right radial artery. We report a 62-year-old woman with coronary artery disease in whom ARSA was not identified preoperatively. During coronary angiography (CAG) performed via right transradial access, iatrogenic dissection of the aberrant right subclavian artery occurred and extended retrogradely to form a Stanford Type B aortic dissection measuring 70 mm in total length, with a 5.6-mm intimal tear at the proximal segment of the ARSA. The patient received conservative management including strict blood pressure control and analgesia after the procedure, and was discharged in improved condition.

## Background

ARSA results from abnormal development of the embryonic fourth aortic arch and is the most common congenital aortic arch anomaly, with an overall prevalence of 0.5%–1% in the general population and a higher prevalence in females (1–4). Multiple congenital thoracic vascular variants including anomalous coronary ostial origins and arteria lusoria, as summarized in recent anatomical reviews, all increase catheterization risks and are aggravated by systemic vessel fragility (5–8). This anomaly cannot be detected by routine examinations such as electrocardiography and echocardiography, and most patients remain asymptomatic throughout life, making it prone to being overlooked.

A normal aortic arch gives off the brachiocephalic trunk, left common carotid artery and left subclavian artery in sequence. As the last branch of the aortic arch, ARSA originates from the distal aortic arch, courses posterior to the esophagus to the right thoracic outlet, and is characterized by tortuosity and sharp angulation (4). Transradial access has become the preferred approach for coronary angiography and interventional therapy owing to fewer puncture-related complications and better patient comfort. However, in patients with ARSA, the congenital anatomical abnormality of the right radial artery makes the vascular intima vulnerable to injury during guidewire and catheter advancement. Previous studies and case reports have demonstrated that right transradial procedures in patients with ARSA may lead to severe vascular complications, including aortic dissection, subclavian artery hematoma, dissection with thrombosis, and acute limb ischemia (9–11).

Iatrogenic Stanford Type B aortic dissection is a rare but life-threatening complication of transradial intervention. Its treatment strategy and prognosis are closely associated with the time of onset, lesion extent, and concomitant visceral or limb ischemia (12). This rare case of iatrogenic dissection of the aberrant right subclavian artery extending to the aorta confirms that this anatomical variant is a high-risk factor for right transradial access. The patient was stabilized after conservative treatment, offering important clinical guidance for risk management in interventional procedures.

## Case report

A 62-year-old female patient was admitted due to intermittent chest pain lasting 7 years, which was unrelated to physical activity. She had a 7-year history of hypertension with a peak blood pressure of 200/110 mmHg. She was treated with nifedipine tablets, yet her blood pressure remained poorly controlled. Long-term antiplatelet therapy with aspirin (Bayaspir®) had been prescribed by physicians at an external hospital. Coronary computed tomography angiography (CCTA) performed at that hospital in 2019 demonstrated 60% stenosis of the left anterior descending artery and 50% stenosis of the right coronary artery. The anomalous right subclavian artery failed to be visualized on the prior CCTA. Physical examination revealed normal cardiopulmonary findings, and bilateral peripheral pulses were symmetrical. All laboratory tests were within normal limits. No significant abnormalities were detected on resting electrocardiography or transthoracic echocardiography. The admission diagnoses included coronary atherosclerotic heart disease, stable angina pectoris, New York Heart Association (NYHA) class II cardiac function, and grade 3 hypertension. The patient presented to our hospital for further evaluation owing to persistent intermittent chest pain despite medication. Her cardiologist recommended coronary angiography (CAG) to precisely characterize coronary lesions. Pre-procedural anatomical evaluation of the aortic arch branches was not performed.

**Figure 1:**
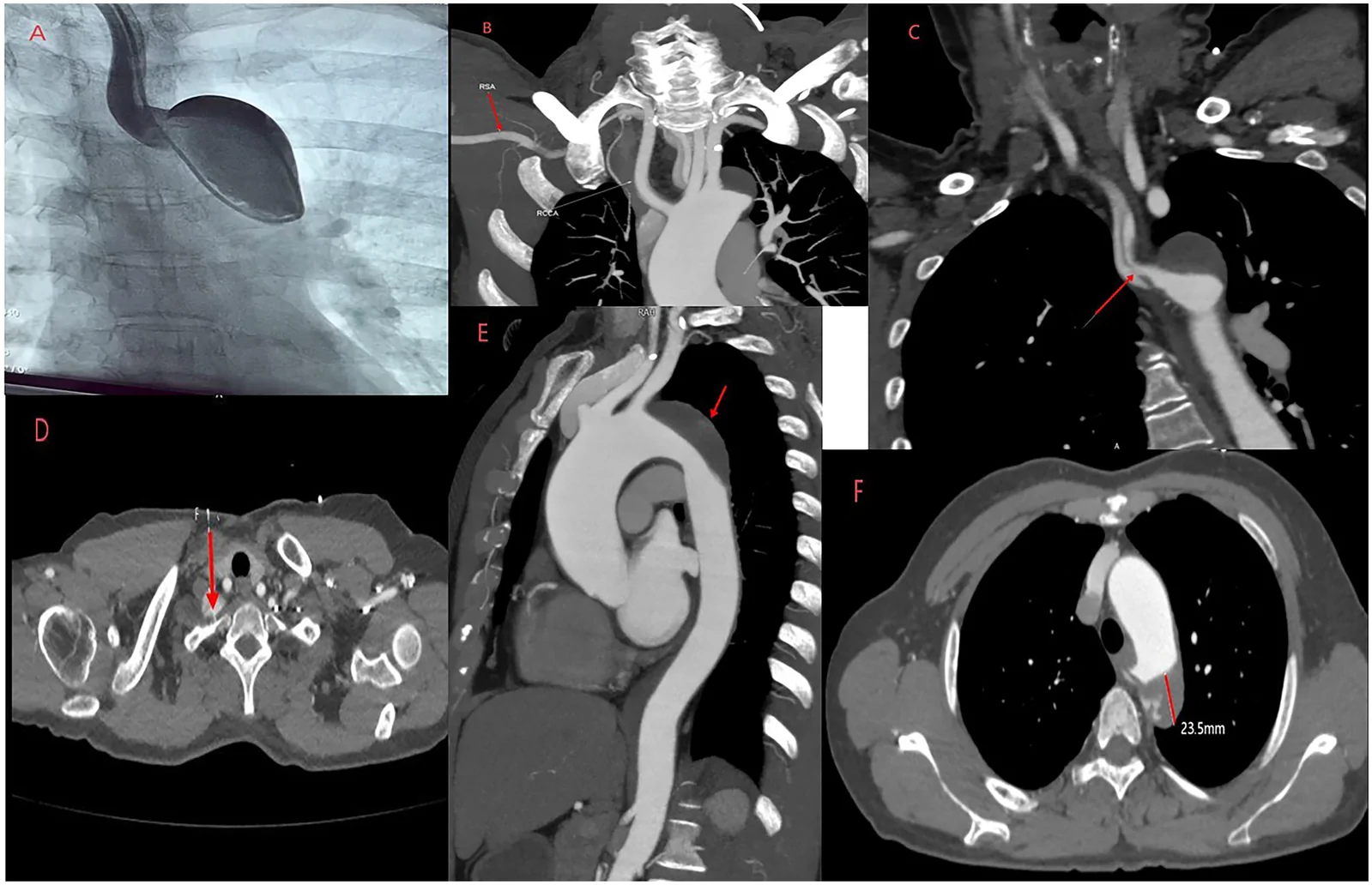
**(A)** CAG shows contrast filling in the false lumen of the distal aortic arch, descending aorta, and brachiocephalic trunk, confirming the diagnosis of aortic dissection. **(B)** Whole-aorta CTA identifies an aberrant right subclavian artery coursing posterior to the esophagus toward the right axilla (arrow). **(C)** CTA demonstrates a dissection lesion (arrow) extending from the proximal right axillary artery to the entire aberrant right subclavian artery. **(D)** A 5.6-mm intimal tear (arrow) is detected at the proximal segment of the aberrant right subclavian artery on CTA. **(E,F)** CTA measurements show that the total length of the aortic dissection is approximately 70 mm. The false lumen achieves a maximum width of 23.5 mm (arrow), with predominant thrombosis formation inside the lumen.

During CAG, significant resistance was encountered when attempting to advance the guidewire into the right subclavian artery, and subsequent catheter manipulation proved difficult. Upon contrast injection after catheter placement, contrast opacification of the false lumen was observed in the distal aortic arch and descending aorta and brachiocephalic trunk (Figure 1A), and the patient complained of back pain. All catheters and guidewires were immediately withdrawn. The patient was closely monitored, and access was converted to the right femoral artery. Coronary angiography was completed uneventfully. No indications for percutaneous coronary intervention were identified. Contrast-enhanced CTA of the entire aorta was performed immediately after the procedure. The aberrant right subclavian artery (ARSA) was identified, which coursed posterior to the esophagus to the right axilla (Figure 1B). A dissection extending from the proximal right axillary artery to the entire ARSA was identified (Figure 1C). An intimal tear measuring 5.6 mm in diameter was detected at the proximal segment of the ARSA (Figure 1D). The dissection extended proximally to the distal aortic arch and descending aorta, consistent with a Stanford Type B aortic dissection (Figure 1E). The total length of the dissection was approximately 70 mm, with a maximum false lumen width of 23.5 mm, most of which was thrombosed (Figure 1F).

**Figure 2:**
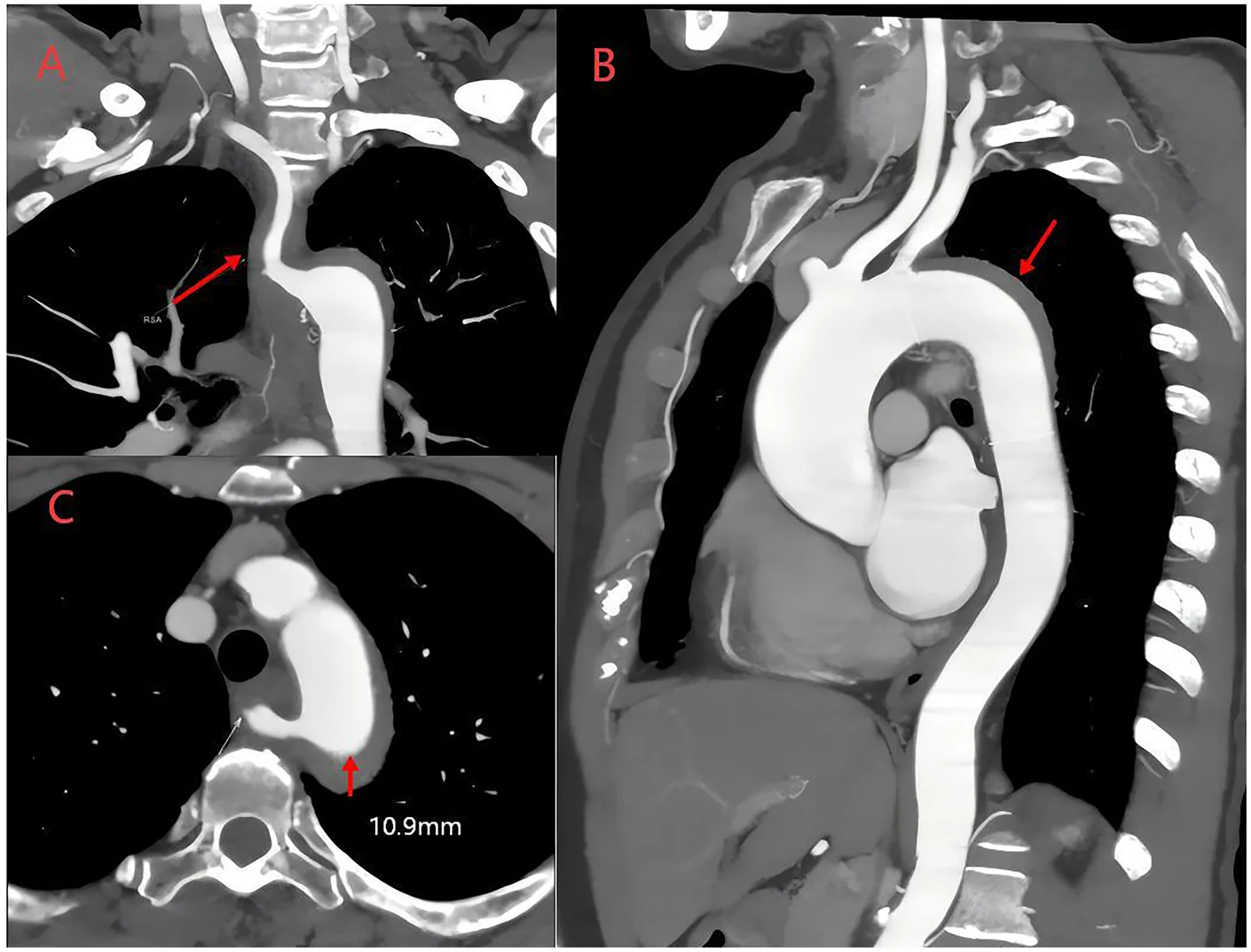
Follow-up CTA images. **(A)** Complete thrombosis of the false lumen in the distal aortic arch, descending aorta, aberrant right subclavian artery (arrow), and right axillary artery. **(B)** Marked reduction in the size of the false lumen (arrow). **(C)** The false lumen has a maximum width of approximately 10.9 mm (arrow).

**Figure 3:**
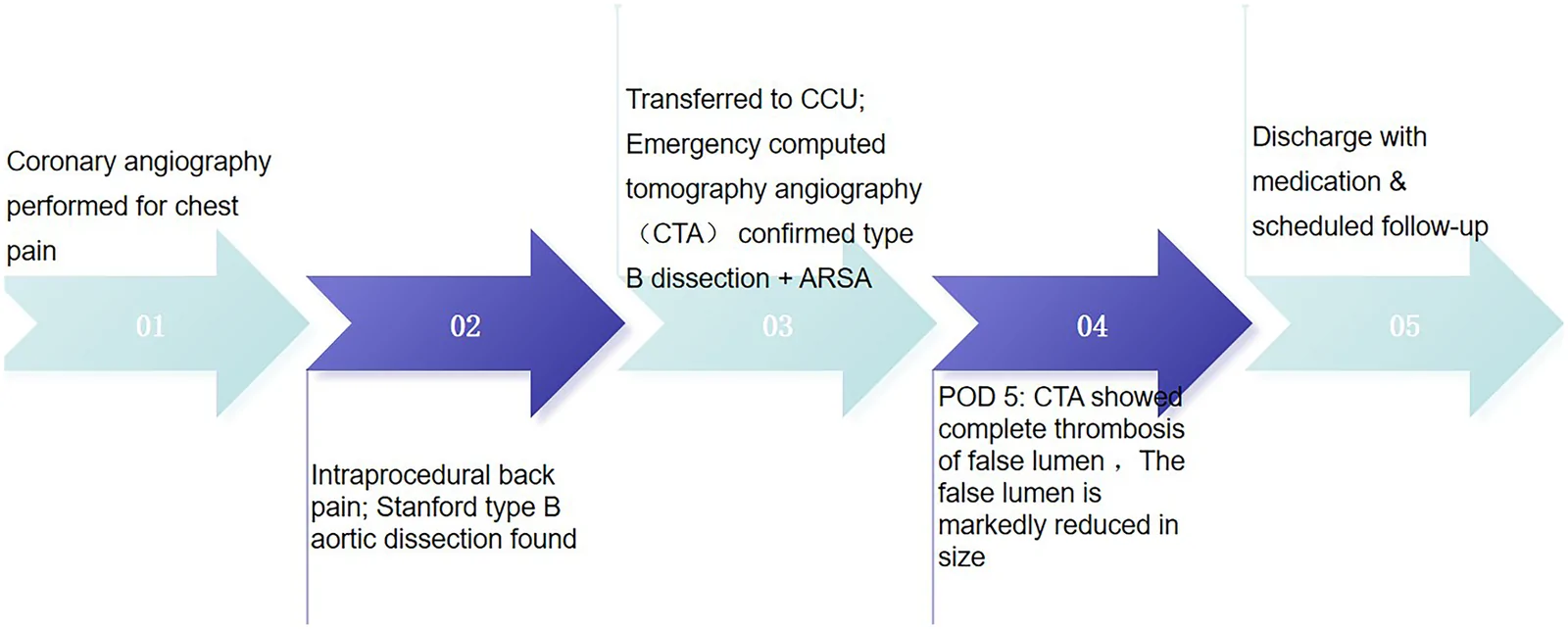
The timeline of the patient's clinical course during hospitalization.

Given the patient's stable vital signs, non-progressive back pain, largely thrombosed false lumen, and the location of the intimal tear at the proximal ARSA, conservative management was pursued. Intravenous beta-blockers and urapidil were administered by continuous intravenous infusion for strict blood pressure control, targeting a systolic blood pressure of <110 mmHg. The patient's pain resolved after analgesia and sedation, and she was closely monitored in the intensive care unit. Follow-up CTA of the great vessels on post-procedural day 5 revealed complete thrombosis of the false lumen in the distal aortic arch, descending aorta, the ARSA, and the right axillary artery. The false lumen in the distal aortic arch and descending aorta had decreased in size, with a maximum width of approximately 10.9 mm (Figures 2A–C). The patient was asymptomatic, with blood pressure well controlled, and was discharged home. Oral antihypertensive therapy consisting of beta-blockers and angiotensin-converting enzyme inhibitors was continued after discharge. Regular outpatient follow-up was recommended, and follow-up aortic CTA scans were scheduled at 1, 3, 6, and 12 months post-procedurally. The timeline of the patient's management during hospitalization is summarized in Figure 3.

## Discussion

This case illustrates that severe complications may occur during transradial coronary angiography in patients with an unrecognized ARSA. The external coronary CTA failed to identify ARSA, as standard coronary CTA protocols focus primarily on coronary artery visualization, yielding limited thin-slice coverage of the entire aortic arch, with reconstruction windows optimized solely for coronary ostial assessment.

In normal anatomy, the right subclavian artery arises from the brachiocephalic trunk with a relatively straight course. As the final branch of the aortic arch, ARSA courses posterosuperiorly with an acute angle at its origin. Catheters must reverse direction to enter the ascending aorta (13, 14). Forceful manipulation during right transradial intervention may easily injure the vessel wall and cause dissection or hematoma. Previous studies have shown a higher access failure rate in patients with ARSA (15–17). Most reported cases involved only access failure without severe adverse events. By contrast, this patient developed iatrogenic aortic dissection, which underscores the potential severity of this complication. The overall incidence of iatrogenic aortic dissection is <0.1% (18). Aggressive catheter manipulation, vascular tortuosity, and underlying aortic diseases are common predisposing factors (19). In this case, the acute angle of ARSA forced the catheter tip against the aortic wall. Long-standing hypertension and atherosclerosis weakened the aortic wall. Additionally, the operator advanced the catheter forcefully instead of withdrawing it to reassess when resistance was encountered. These factors collectively led to dissection.

This case demonstrates that unrecognized anatomical variants encountered during transradial catheterization carry comparable procedural risks, and such complications are not isolated incidents. As outlined by Gaydarski et al., arteria lusoria (aberrant right subclavian artery) creates mechanical friction and directional catheter conflict absent in the standard three-vessel aortic arch anatomy; this mechanical stress synergizes with pre-existing vascular fragility to trigger vessel dissection (5). Such fragility may stem from chronic hypertensive arteriosclerosis, as observed in our patient, or systemic inflammatory connective tissue disease, which was validated by Stoimenov et al. in their cohort of patients with ankylosing spondylitis and diffuse aortic wall degeneration (6). Coronary anatomical variants confer identical mechanistic hazards: anomalous coronary ostial origins distort catheter alignment and generate shear stress that damages the intimal layer during guidewire advancement. Dimitrova et al. reported isolated coronary ostial malformations without concomitant aortic arch anomalies that still resulted in endothelial injury during routine angiography, verifying that all congenital vascular deviations—whether involving the aortic arch or coronary root—independently or cumulatively elevate interventional risks (7, 8). Collectively, pre-procedural screening for all congenital thoracic vascular variants, rather than only arteria lusoria (ARSA), is critical for mitigating the risk of iatrogenic aortic trauma.

Complete thrombosis and marked shrinkage of the false lumen were observed on post-procedural day 5 in this case. Among previously reported secondary aortic dissections induced by transradial catheterization, partial and slowly progressive thrombosis of the false lumen typically develops after a minimum of 10–14 days under similarly strict conservative antihypertensive regimens (9, 10), making our finding relatively rare. The accelerated thrombotic response in our patient may be attributed to the dissection tear being confined to a short proximal segment of the aberrant right subclavian artery, which limits the extent of persistent false lumen perfusion from the true aortic lumen. Early and rapid thrombosis confers a favorable prognosis and reduces long-term risks of aneurysmal dilation or delayed aortic rupture.

## Conclusion

This case has several clinical implications. For patients scheduled for right transradial coronary angiography, clinicians should carefully review chest radiographs or chest CT scans before the procedure. Further aortic CTA imaging is recommended if an abnormal aortic arch position or mediastinal contour is found, to exclude anatomical variants. During the procedure, difficult catheter advancement due to vascular tortuosity in the subclavian region should raise suspicion of ARSA. Operators may use softer and more flexible guidewires, or switch to alternative access sites such as the contralateral radial artery or femoral artery. Management of iatrogenic aortic dissection depends on lesion extent and hemodynamic status. Stanford Type A dissection generally requires emergency surgery (20). Stanford Type B dissection is primarily managed with medication, unless malperfusion or rupture occurs (21). This patient had Stanford Type B aortic dissection with stable vital signs and spontaneous thrombosis of the false lumen, prompting the adoption of conservative management. Given the inherent limitations of a single-case report and the limited follow-up duration, these findings cannot capture the full spectrum of clinical features and long-term outcomes of such conditions.

Written informed consent was obtained from the patient for the publication of this case report and all accompanying images.

## Data Availability

The original contributions presented in the study are included in the article/Supplementary Material, further inquiries can be directed to the corresponding authors.
